# PD132 A Target Trial Emulation Application Assessing The Survival Of Erythropoiesis-Stimulating Agents In Patients With Lower Risk Myelodysplastic Syndrome

**DOI:** 10.1017/S0266462324003696

**Published:** 2025-01-07

**Authors:** Alastair Bennett, Andrea Manca

## Abstract

**Introduction:**

Patients with myelodysplastic syndromes (MDS) can be treated with erythropoiesis-stimulating agents (ESAs) to alleviate anemia-related symptoms and delay the need for expensive transfusions. However, clinicians disagree on prescribing ESAs because evidence on the effectiveness of ESAs is limited. This study aimed to reliably estimate the survival of a dynamic ESA treatment regimen using a novel causal inference approach.

**Methods:**

The European MDS Registry collects data on patients with MDS every six months. We followed a two-step framework to develop a hypothetical and emulated trial protocol. The eligible population consisted of patients with intermediate-1 to low-risk MDS who were treatment-naïve, had a hemoglobin concentration of less than 10 grams per deciliter, and did not have a chromosome 5q deletion (non-del(5q) MDS). Red blood cell transfusion was allowed before the date of diagnosis. Patients were cloned and assigned to both treatment groups, thereby eliminating immortal time bias, and were censored as soon as they stopped following the assigned treatment strategy. This artificial censoring introduced selection bias, which was adjusted for by using inverse probability of censoring weighting. The weights model adjusted for time varying confounders.

**Results:**

Of the 611 patients qualifying for the study, 282 started ESAs within the six-month grace period and 329 did not take ESAs. The median follow-up was 2.4 years (interquartile range 1.3 to 4.2). A naïve analysis of our cohort suggested that no ESA was significantly more beneficial than taking ESAs (hazard ratio [HR] at year four: 1.24, 95% confidence interval [CI]: 1.03, 1.50). However, after correcting for biases the adjusted Kaplan-Meier curves showed that ESAs were beneficial over the first two years (HR at year one: 0.75, 95% CI: 0.41, 1.39), compared with no ESAs. Thereafter there was no difference between treatment groups (HR at four years: 1.01, 95% CI: 0.80, 1.27).

**Conclusions:**

We found that early—within six months of becoming eligible—initiation of ESAs as first-line therapy for treatment-naïve patients with non-del(5q) low-risk MDS and hemoglobin levels of less than 10 grams per deciliter improves survival over for the first two years. Using target trial emulation to make accurate survival estimates can improve decision-making in health technology assessment.

